# Role of Periodontal Bacteria, Viruses, and Placental *mir155* in Chronic Periodontitis and Preeclampsia—A Genetic Microbiological Study

**DOI:** 10.3390/cimb43020060

**Published:** 2021-07-29

**Authors:** Jaideep Mahendra, Little Mahendra, Maryam H. Mugri, Mohammed E. Sayed, Shilpa Bhandi, Rahaf Turki Alshahrani, Thodur Madapusi Balaji, Saranya Varadarajan, Swetha Tanneeru, Abirami Nayaki Rao P., Sruthi Srinivasan, Rodolfo Reda, Luca Testarelli, Shankargouda Patil

**Affiliations:** 1 Department of Periodontology, Meenakshi Ammal Dental College and Hospital, Chennai 600095, India; abirami.nayaki1791@gmail.com (A.N.R.P.); drsruthisvasan@gmail.com (S.S.); 2 Department of Periodontology, Maktoum Bin Hamdan Dental University College, Dubai 213620, United Arab Emirates; littlemahendra24@gmail.com; 3 Department of Maxillofacial Surgery and Diagnostic Sciences, College of Dentistry, Jazan University, Jazan 45412, Saudi Arabia; dr.mugri@gmail.com; 4 Department of Prosthetic Dental Sciences, College of Dentistry, Jazan University, Jazan 45412, Saudi Arabia; drsayed203@gmail.com; 5 Department of Restorative Dental Sciences, College of Dentistry, Jazan University, Jazan 45412, Saudi Arabia; shilpa.bhandi@gmail.com; 6 College of Dentistry, Jazan University, Jazan 45412, Saudi Arabia; dr.rahafturki@gmail.com; 7 Tagore Dental College and Hospital, Chennai 600127, India; tmbala81@gmail.com; 8 Department of Oral Pathology and Microbiology, Sri Venkateswara Dental College and Hospital, Chennai 600130, India; vsaranya87@gmail.com; 9 Department of Periodontics, Narayana Dental College and Hospital, Nellore 534003, Andhra Pradesh, India; swethatanneeru@yahoo.com; 10 Department of Oral and Maxillo Facial Sciences, University of Rome La Sapienza, 00161 Rome, Italy; rodolforeda17@gmail.com (R.R.); luca.testarelli@uniroma1.it (L.T.); 11 Division of Oral Pathology, Department of Maxillofacial Surgery and Diagnostic Sciences, College of Dentistry, Jazan University, Jazan 45412, Saudi Arabia

**Keywords:** chronic periodontitis, *mir* 155, periodontal microflora, preeclampsia, pregnancy

## Abstract

Previous studies assessed the involvement and impact of periodontal bacteria in preeclamptic women with chronic periodontitis. To explore further, the current study aimed to associate periodontal viruses and bacteria with *mir155* levels in placental tissues of preeclamptic women with generalized chronic periodontitis. Four-hundred 45 pregnant women, 18–35 years of age, were selected and divided into four groups (controls, A, B, and C) where the Controls included 145 systemically and periodontally healthy pregnant women Group A-100 systemically healthy pregnant women with chronic periodontitis, Group B- 100 preeclamptic women with chronic periodontitis, Group C- 100 preeclamptic women without chronic periodontitis. Age, BMI, SES, and periodontal parameters such as PI, BOP, PPD, and CAL were noted. Periodontal pathogens such as *Tf, Td, Pg, Pi, Fn*, HSV, EBV, and HCMV were tested in subgingival plaque, placental tissues, and mir155. We observed that PI, BOP, PPD, CAL, *Tf,* and EBV were highly significant in Group B. We found a higher number of periodontal bacteria, viruses, and *mir* 155 in Group B showing a higher risk of preeclampsia. More genetic studies in this field are advised to ascertain the role of periodontopathogens and *mir* 155 in preeclampsia and periodontal inflammation. What is already known on this subject? Periodontal diseases pose an increased risk of developing preeclampsia and delivering preterm and/or low-birth-weight babies. What do the results of this study add? Periodontal variables such as PI, pocket depth, BOP, and clinical attachment levels, were found to be increased in the preeclamptic women with chronic periodontitis. The significant difference was seen in the relative fold expression of mir155 with higher gene expression of mir155 in groups B and A as compared to group C and controls. What are the implications of these findings for clinical practice and/or further research? In our study, *mir*155 correlation with the periodontal parameters and periodontal pathogens further strengthen the evidence of periodontal inflammation as a risk of preeclampsia in pregnant women especially when associated with chronic periodontitis. *mir*155 can be considered to be one of the genetic biomarkers and can be used as a diagnostic tool for the early detection of PE.

## 1. Introduction

Chronic periodontitis is an infectious disease resulting in inflammation of the supporting tissues of the teeth, progressive attachment, and bone loss and is characterized by pocket formation and/or recession of the gingival and recognized as the most frequent form of periodontitis [1]. The disease is multifactorial in origin and the tissue destruction associated with chronic periodontitis is believed to be the result of host-microbial interaction. Progression of tissue loss usually occurs slowly, but periods of rapid progression can also occur depending on host susceptibility and microbial virulence [2]. Dental plaque is a complex biofilm mainly composed of anaerobic bacteria such as *Porphyromonas gingivalis(Pg)*, *Tannerella forsythia(Tf)*, *Treponema denticola(Td)*, *Prevotella intermedia(Pi)*, *Fusobacterium nucleatum(Fn)* which play a crucial role in the periodontal disease pathogenesis [2]. Recently, viruses such as Herpes Simplex Virus (HSV), Epstein Barr Virus (EBV), Human Cytomegalovirus (HCMV) have shown to be implicated in periodontal diseases, contributing to the etiopathogenesis of periodontitis in bacterial association [3]. Evidence suggests that these microorganisms or their products have a deleterious effect on the host either directly or indirectly by stimulating host tissues resulting in the release of inflammatory cytokines [3,4].

Microbial dissemination during periodontal infections has been associated with various systemic illnesses such as preterm-low-birth weight (PTLBW) and preeclampsia [5]. Preeclampsia (PE) is a pathological condition characterized by hypertension and proteinuria seen in pregnancy. It is said that bacteria and their antigens cross the placental barrier through the bloodstream and reach the uterine cavity, which might lead to adverse pregnancy outcomes [4]. Studies have postulated that periodontal diseases are at increased risk of developing preeclampsia and delivering preterm and/or LBW babies [5,6]. Studies also demonstrate the role of periodontal treatment in reducing the risk of adverse pregnancy outcomes as well [7,8,9]. Micro RNA 155 is believed to play a key role in modulating humoral and innate cell-mediated immune responses. It is crucial for proper lymphocyte development and maturation and is closely related to the presence of inflammation [10]. A broad range of viral and bacterial inflammatory mediators can stimulate the expression of *mir*-155-5 p indicating the intimate relationship between inflammation, innate immunity, and *mir155 hg* expression. It was also found that overexpression of *mir*-155 might lead to a chronic inflammatory state in humans [11].

Thus, the present research was designed to assess demographic variables, periodontal parameters, and periodontal microorganisms (bacteria and viruses) in dental plaque and samples from the placenta of preeclamptic pregnant women with and without chronic periodontitis. *mir155* levels were also estimated in placental samples in all the groups.

## 2. Materials and Methods

**Figure 1:**
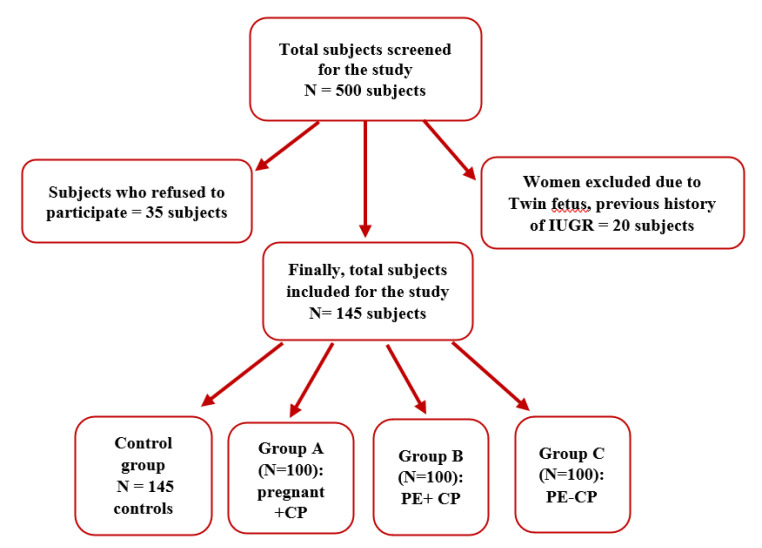
Study flow chart.

Five hundred pregnant women aged 18–35 years from Obstetrics and Gynaecology Department, Narayana Medical Hospital, Nellore, Andhra Pradesh, India were screened during the period August 2015 to August 2018. Of these, 35 individuals refused to participate in the study, and 20 were excluded due to twin fetuses, previous history of intrauterine growth restriction (IUGR), and uterine infections. Finally, 445 pregnant women were recruited and were categorized into four groups based on inclusion and exclusion criteria. Controls–145 healthy (systemic + periodontal) pregnant women, Group A–100 Systemically healthy pregnant women with chronic periodontitis (CP), Group B–100 Preeclamptic pregnant women with chronic periodontitis (PE + CP), Group C–100 Preeclamptic pregnant women without chronic periodontitis (PE) (Figure 1). The power of the study was calculated to be 95%. The systemic health status of the participants was obtained from previous medical records. The diagnosis of pre-eclampsia was given by a certified.

### 2.1. Inclusion Criteria

The study included pregnant women between the ages of 18 and 35 who had a single pregnancy, had at least 20 natural teeth, had not received periodontal therapy in the previous six months, were not using any mouthwashes, and were willing to participate.

### 2.2. Exclusion Criteria

The study excluded pregnant women who were currently using any kind of cigarettes, had a history of alcohol intake or were using systemic drugs and with history of any other systemic illnesses such as diabetes, liver diseases, renal diseases, arthritis, cardiovascular diseases, epilepsy, HIV infection, and respiratory infections.

The Institutional Ethical Board (RC.no.NDC/staff/2015-16/EC/2015/01) Narayana Dental College and hospital, Nellore, Andhra Pradesh, India, approved the study. Written informed consent was obtained from all participants who were enrolled after explaining the nature of the present study. The study was registered with Clinicaltrials.gov (NCT04277390) and was conducted following the Helsinki Declaration of 1975, as revised in 2013. Demographic information collected included: (1) Age, (2) Body mass index (BMI), (3) Socioeconomic status (SES), (4) Blood pressure and (5) Urine protein content. Information regarding gestational age, trimester, previous pregnancies, medical and dental history was also obtained.

### 2.3. Clinical Examination

The periodontal examination was done by a single investigator. Clinical parameters recorded were plaque index (PI) [11] (2) bleeding on probing (BOP) [11] (3) probing depth (PD) [12] and (4) clinical attachment loss (CAL) [12] using a Williams periodontal probe. The Bleeding on probing (%), PPD and clinical attachment loss (mm) was calculated as a mean of all sites of the entire dentition. Participants were diagnosed with chronic periodontitis if they had: (1) four or more teeth with PD ≥ 4 mm at one or more sites; (2) CAL ≥ 3 mm at the same site with BOP.

### 2.4. Subgingival Plaque Collection

Subgingival plaque samples were collected during the second trimester from all the participants (N = 445) with the help of a sterile curette and stored in sterile tubes in −80 °C for microbial analysis.

* TRIzol reagent, catalog#15596026, Thermo Fischer scientific Inc., Waltham, MA, USA.

### 2.5. Collection of Placental Tissue Samples

Placental tissue samples were obtained using a surgical blade from the middle zone of the placenta after removing amniotic and decidual membranes with the help of a. Samples were cleaned with distilled water, placed in sterile tubes containing RNA stabilizing reagent *, and further stored at −80 °C for molecular analysis.

### 2.6. Molecular Analysis

Subgingival and placental tissue samples were subjected to a real-time polymerase chain reaction for quantification of periodontal microorganisms (*Tanerella forsythis(Tf), Treponema denticola(Td), Prevotella intermedia(Pi), Porphyromonas gingivalis(Pg), Fusobacterium nucleatum(Fn)* Herpes simplex virus (HSV), Ebstein Barr Virus (EBV), and Human cytomegalovirus (HCMV)). Expression levels of Micro RNA 155 (*mir155*) were also assessed in all groups.

### 2.7. Isolation of DNA from Both Subgingival and Placental Tissue Samples

Plaque DNA was extracted by standard laboratory phenol/chloroform extraction method by modifying Stephen et al. method [13]. Similarly, placental tissue was homogenized using a tissue homogenizer. Both were incubated with 100 µL of lysis buffer with 10% Sodium dodecyl sulfate solution *(*SDS) and 5 µL of Proteinase-K for digestion at 37 °C for 1 h. After disruption, 1 mL phenol-chloroform (3:1 volume/volume) was added and samples were centrifuged for 10 min at 10,000 rpm. A second phenol/chloroform extraction was performed after collecting the supernatant. The samples were allowed to stand at −20 °C for 2 h and DNA was precipitated with 0.5 mL isopropanol. The product was then centrifuged at 10,000 rpm for 15 min, the supernatant was discarded and the aliquots were washed in 0.5 mL of 70% alcohol, dissolved in 0.2 mL sterile nuclease-free milli-Q water. DNA concentration was then measured using spectrophotometer ^†^ at 260/280 nm. The product was stored at −20 °C for further microbial analysis.

1. † NANODROP, Thermo Scientifics, Waltham, MA, USA
2. ‡ BIORAD-CFX100 (BIORAD, Hercules, CA, USA)

### 2.8. Pcr Procedure for Bacterial and Viral Identification

#### Red Complex Bacteria and Viruses

Quantitative RT-PCR was performed with the real-time thermocycler. ^‡^ The primers of red complex bacteria and viruses were designed and by NCBI-BLAST search using the base of 16 S rRNA gene and synthesized. ^§^ The double-standard DNA-binding dye SYBR Green^‖^ was used in RT-PCR for the detection of microbial DNA. Species-specific primers were used for *Pg, Tf, Td, Pi*, *Fn*, HSV, EBV, and HCMV and the reaction efficacy was optimized. (Table 1) PCR conditions for bacteria and viruses were set up based on manufactures instructions. ^§^ (Table 2 and Table 3) Melt curve analysis was performed for each sample to determine the presence of nonspecific products, multiple amplicons, and contaminants. The comparative cycle threshold units (CT) method was used to calculate the number of genes. The quantification of red complex bacteria and viruses was expressed in CT. The relative quantification of the bacterial and viral count was achieved by compared to the standard amplification curve obtained from the (standard) genomic DNA. The Biorad CFX 96 software computed the CT values that were compared with the CT inferred from the amplification curve of a standard sample containing genomic DNA equivalent to 6 × 10^6^ CFUs of each microorganism.

PCR products were visualized using 2% agarose gel electrophoresis and 100 bp molecular marker DNA and were visualized at the wavelength of 260 nm, ultraviolet transilluminator in the gel documentation system. ^¶^ The mix was prepared in hard-shell 96 well-PCR plate ^#^ with seal plates of optically transparent film ** Care was taken to seal the plate edges and corners to prevent artifacts caused by evaporation. The amplification was performed in a real-time thermocycler ^††^. SYBR Green” channel of the real-time instrument (BIORAD-CFX100) was used for the quantification using Luna Universal Master Mix. ^‡‡^

1. § Bioserve Biotechnologies Pvt. Ltd. Hyderabad and IRA biotechnology Pvt. Ltd. Hyderabad, India.
2. ‖ LUNA UNIVERSAL Qpcr master mix Kit-NewEngland Biosciences, MA, USA.
3. ¶ Syngene International Limited, Bengaluru, Karnataka, India.
4. # Bioserve Biotechnologies Pvt. Ltd. Hyderabad and IRA biotechnology Pvt. Ltd. Hyderabad, India.
** cat.no. HSP 9601 (Bio-Rad Laboratories Inc., Hercules, CA, USA.)
6. ^††^ Bio-Rad Laboratories Inc., USA.
7. ‡‡ Thermocycler, BIORAD-CFX100, BIORAD, Hercules, CA, USA.

### 2.9. Estimation of mir155 Levels in Placental Samples

Placental samples were thawed at room temperature. Extraction of total RNA was done using guanidium thiocyanate (GITC) [13]. Tissues were washed initially using saline and then lysed in solution D.^§§^ water-saturated phenol precipitation was used to separate the RNA-containing aqueous phase. After an ethanol wash, the RNA was precipitated with isopropyl alcohol and air-dried. Finally, the RNA pellet was dissolved in RNAse free water and stored at −20 °C for further use. Purity check of *mir* 155 purity was done using NANODROP reading at 260/280 nm. Complementary DNA (cDNA) for miRNAs was prepared using a universal stem-loop primer (USLP). The cDNA samples were stored at −20 °C for further quantification of *mir* levels using sequence-specific primers.

### 2.10. Real-Time Quantitative PCR (Rt-Qpcr) for Analyzing mir-155 Expression Levels

A 15 min of melt in the f-curve analysis was performed following the reaction cycles to differentiate amplicons from primer-dimer. Each reaction was performed in triplicates to avoid technical errors during the RT-qPCR reaction set up. *mir155* expression levels were assessed by SYBR Green-based RT-qPCR through relative quantification by normalization with endogenous control U6. Ct values for all miRNAs in each sample were recorded and used to calculate fold change compared to their endogenous controls.

### 2.11. Statistical Analysis

The data were entered in MS-Excel and statistical analysis was done using IBM statistical package for Social Science SPSS (Version 24.0) for Microsoft windows. For categorical variables, the data values were represented as number and percentages. For continuous variables, the data values were shown as mean and standard deviation (SD).

§§ Solution D catalog # K1156, Thermo Fischer scientific Inc., Waltham, MA, USA.

ANOVA test with post hoc (Tukey’s HSD) test was used for analyzing demographic variables, periodontal variables, and Microorganisms (*Tf, Td, Pi, Pg, Fn,* HSV, EBV, HCMV) for testing the mean difference between four groups.

One-way ANOVA was used to compare the *mir155* expression and its significance among groups. *p* values with less than 0.05 were considered statistically significant. Fold change values of *mir155* were calculated using the 2^−ΔΔCt^ method of relative quantification. A regression value of ≥0.99 was considered statistically significant with 100% PCR efficiency. All the statistical analysis was performed and represented using Graph Pad Prism software. ^‖‖^

## 3. Results

On comparing demographic variables such as mean age, mean BMI, and mean socio-economic status, no statistically significant difference was found between groups. However, mean Systolic blood pressure (SBP), mean Diastolic blood pressure (DBP), and mean urine protein content were higher and found to be statistically significant among groups B and C when compared to controls and group A participants. (*p*-value < 0.0001) (Table 4).

Periodontal parameters such as PI, PPD, BOP, and CAL, were found to be greater in the preeclamptic women with chronic periodontitis (group B) compared to the other groups. (*p*-value < 0.0001) (Table 4) *Tf, Td, Pi, Pg, Fn* counts were also found to be high in group B than A, C, and control groups in both subgingival plaque and placental tissue samples. (Table 5 and Table 6) Similarly, viral counts were also higher in group B compared to groups A, C, and controls in both subgingival plaque and placental tissue samples (Table 5 and Table 6).

**Figure 2:**
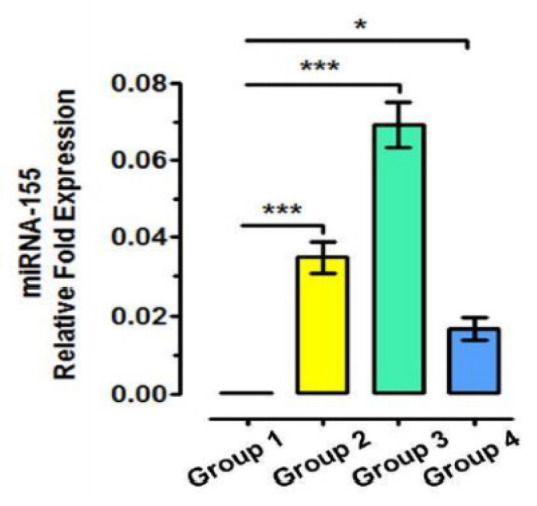
Relative fold expression of MIR155 among groups. Controls: 145 Systematically and periodontally healthy pregnant women. Group A: 100 Systematically healthy pregnant women with chronic periodontitis. Group B: 100 Preeclamptic pregnant woman with chronic periodontitis. Group C: 100 Preeclamptic pregnant woman without chronic periodontitis. * statistically significant, *** highly statistically significant. ‖‖ Version V, SanDiego, CA, USA.

The significant difference was seen in relative fold expression of *mir155* among groups (*p*-value < 0.0001) with higher gene expression of *mir155* in groups B and A as compared to group C and controls. (Figure 2).

On correlating *mir155* with periodontal parameters and microorganisms, a positive correlation was found with BOP, PPD, and HSV in controls. In group A, a positive correlation was seen in HSV alone. In group B, a positive correlation was observed with PPD, CAL, *Fn,* and HSV, while in group C, a positive correlation was expressed with PI, *Fn,* and HSV (Table 5 and Table 6).

## 4. Discussion

Preeclampsia (PE) is a pregnancy-specific disorder characterized by hypertension and proteinuria affecting 5–10% of all pregnancies [14]. Even though the exact etiology of PE is not well known, this syndrome is thought to be the result of inflammatory endothelial dysfunction and is said to involve poor placental invasion, placental inappropriate development, and placental oxidative stress [14]. Periodontal disease, on the other hand, is a chronic inflammatory disease initiated in response to Gram-negative, anaerobic, subgingival bacteria. Even though chronic periodontitis starts as a local inflammation of periodontal tissues, it is said that immunological changes that resulted due to periodontal infection can direct the development of PE [14]. Various studies have stated that the periodontal inflammatory process can contribute to extra-oral pathologies such as cardiovascular diseases and preeclampsia [5,6,14,15,16].

Preeclampsia is a chronic inflammatory condition involving various risk factors. The previous literature has suggested periodontitis as a risk of preeclampsia [5,6,14,15,16]. In the past, periodontal bacteria have been associated with preeclampsia; however, the viral etiology concerning this disease is still unknown. Our study hypothesizes the integrated role of the bacterial-viral complex in the progression of preeclampsia. In the present study, we aimed to quantify periodontal bacteria and viruses in both dental plaque and placenta of preeclamptic women with periodontitis. Interestingly, *mir*155 levels were also estimated in placental tissues postpartum.

In the present study, demographic variables such as age, BMI, and SES were similar among all the participants, indicating that all participants were sharing similar demographic characteristics. In our study, the age range of all the participants was between 18–35 years. This was similar to a study done by Mahendra et al. in 2016, where the authors compared age, BMI, and SES in preeclamptic and normotensive women which were found to be similar. Advanced maternal age itself is a risk factor for PE. The authors suggested that this risk might be due to relative aging of the uterine blood vessels further leading to altered vascular response with the advance in age [17]. Morteza Motedayen et al. [18] revealed a significant relationship between BMI and risk of preeclampsia, which could be attributed to the altered hormonal changes in women with high BMI.

Socioeconomic status was found to be similar among all the participants in the present study, which demonstrates that the selected participants were relatively homogeneous due to study randomization. Silva et al. [19] showed that pregnant women with low SES had a five-fold increased risk of preeclampsia compared to women with high SES and explained that this risk could be due to low education, financial difficulties, occupational exposure to prolonged walking, driving, and BMI.

SBP, DBP, and urine protein levels were all greater in Group B (PE + CP) than in the other groups in our study. This was similar to a study by Ben-Juan Wei et al. [5] who also stated increased blood pressure in preeclampsia. Usually, PE women will represent higher systolic and diastolic blood pressure due to endothelial cell dysfunction which is a key feature in preeclampsia therefore enhancing the induction of proinflammatory cytokines which in turn leads to oxidative stress injuring endothelial cells. The authors stated that free radicals of oxygen can stimulate lipid peroxidation of free fatty acids under hypoxic conditions leading to the injury of endothelial cells [20]. Beck and Offenbacher [21] also reported that periodontal disease acts as a vascular stressor.

In the present study, urine protein levels were higher in preeclamptic women compared to other groups (Table 4). Sugimoto et al. [22] demonstrated that glomerular endothelial cell detachment and hypertrophy was due to anti-VEGF antibodies and sFlt-1 which in turn resulted in down-regulation of nephrin.

In the present study, PI, PPD, BOP, and CAL were found to be higher in Group B (PE + CP) compared to other groups (Table 4). These findings were consistent with the findings of Girija et al. [14] who also showed the higher periodontal parameters in the preeclamptic group compared to their healthy peers. The authors suggested that inflammatory challenge during preeclampsia might aggravate the overall inflammatory process and might have resulted in additional periodontal destruction in preeclamptic women compared to women in other study groups [14].

Immune responses play a major role in pregnancy to maintain a healthy equilibrium for both mother and the developing fetus. The rise in the number of pro-inflammatory cytokines might pose an increased risk of tissue destruction in periodontal tissues for the existing microbial challenge [15].

In our study, both subgingival plaque and placental tissue samples showed higher levels of *Tf, Pg, Fn*, *Pi,* and *Td* (Table 5 and Table 6) in Group B (PE + CP) participants compared to group A, C, and controls. Our study was consistent with Barak et al. [5], who showed significantly higher bacterial counts (*Aa, Fn*, *Pg, Tf, Td, P*i) and their products in human placental samples of preeclamptic women and suggested that periodontal pathogens play a key role in the pathogenesis of PE. The role of bacterial infection in the development of adverse pregnancy outcomes is well known [7,8,23]. The link between chronic periodontal disease and pregnancy complications might be due to repeated exposure of periodontal bacteria into the uterine cavity through transient bacteremia [4,5,8]. Han et al. [24] also showed hematogenous spread of *Fn* into the placenta resulting in adverse pregnancies in murines. *Fn* is associated with both periodontal disease and adverse pregnancy complications [25]. It is suggested that *Fn* in the oral cavity may spread hematogenously to infect the pregnant uterus [26]. *Fn* has shown to induce FadA adhesion which mediates cell-to-cell epithelial and endothelial attachments therefore playing a pivotal role in intrauterine infection [27]. *Pg* an important periodontal microbe has also been identified in the amniotic fluid of women who are at risk of premature delivery and in the placentas of PE patients [28]. Animal studies have also shown the ability of *Pg* to pass through the placenta, resulting in chorioamnionitis and placentitis [29,30].

It was thought that the uterine cavity and placenta were sterile, but recent advances proposed that the uterine cavity has a unique microbiome that represents a balanced microbial community and was responsible for maintaining a healthy environment. Any dysbiosis in such communities direct to the development of inflammation and contributes to adverse pregnancy outcomes such as PTLBW and IUGR. Any infectious ignite could trigger an inflammatory response in the body and contribute toward PE. There is increasing evidence of viral etiology in periodontitis. Contreras et al. suggested that the herpes viruses play a crucial role in periodontal pathogenesis. Periodontal viruses such as CMV, HSV, and HCMV are ubiquitous and can persist in the host cells of the immune system even after primary infection and were said to be distinct microbial etiologic agents in the development of periodontitis [31]. It is suggested that HSV, EBV, HCMV are the three significant viruses involved in the pathogenesis of periodontitis however the role of the same has not been elucidated in adverse pregnancy outcomes. Hence in our study, we intended to quantify the periodontal viruses keeping in mind the role of bacteria-virus interplay in inflammatory diseases such as preeclampsia which has not been explored so far. In the present study, the periodontal viruses were found to be significant in group B compared to the other groups (Table 5 and Table 6).

Similarly, few studies reported that EBV and HCMV were significantly associated with increased risks of chronic and aggressive periodontitis [32,33]. It is said that bacterial and viral infections in pregnancy can induce the release of pro-inflammatory cytokines such as TNF-ɑ, IL-12, and IFN-γ which elevate oxidative stress and result in endothelial cell dysfunction, which all together lead to PE [34]. There is a possibility that viruses and bacteria together act synergistically in periodontal pathogenesis [35]. It is stated that HSV may produce pathology in the host either directly, indirectly modulating the immune responses [36]. Local immune modulation carried out by these viruses could make it easy for bacterial proliferation, or might increase the bacterial virulence on the host which in turn leads to the release of inflammatory cytokines from the local cells which would lead to further tissue destruction [36]. Gao et al. [37] also proved that EBV is one of the important viruses associated with the progression of periodontitis with an odds ratio of 6.199. CMV was detected positively in periodontitis patient’s subgingival plaque.

Micro RNAs play an important role in several processes and would correlate many pathologies such as PE [38]. *mir*155 is processed in humans from exon 3 of the non-protein-coding B-cell integration cluster (BIC) RNA. Activated B-cells and T-cells, induce its expression. *mir*-155 is thought to enhance inflammatory pathways that are mediated through Activator protein *1**/***Nuclear Factor kappa-light-chain-enhancer of activated B-cells (AP-1/NF-kB) (Bounds et al. 2017). In the present study, elevated levels of placental *mir155* were seen in the Group B (PE + CP) patients than in other groups (Figure 2). This was consistent with the results of a study that showed increased *mir*-155 expression in women with shallow placental invasion seen in PE [38]. Yang et al. [39] also found elevated levels of *mir*-155 in placentas and serum of women with late-onset of PE. O’Connell et al. [40] also found *mir*-155 as a common target in inflammatory conditions.

Many studies have reported the multifactorial role of periodontal pathogens and biomarkers in PE [5,14,15,23,24,25,36]. Lu et al. [41] studied *Pg, Aa, Fn, Pi, Tf, Td*, EBV, HCMV, and HSV in salivary samples of pregnant women with and without chronic periodontitis and found higher counts of EBV and *Pg* in pregnant women with periodontitis compared to controls. The authors stated that EBV and *Pg* co-infection might promote periodontal pathogenesis among pregnant women [41].

Although the current study might not clearly explain the disease-causing relationship between viral (EBV, HCMV, HSV) and periodontal bacterial (Pg, Pi, Tf, Td, Fn) co-infection in preeclampsia, it suggests the role of viral-bacterial interplay in the pathogenesis of preeclamptic pregnant women with chronic periodontitis.

To the best of our knowledge, no studies so far have correlated the placental *mir155* levels with periodontal status, pathogenic bacteria, and viruses. Keeping the association of *mir155* and tissue inflammation in mind, we attempted to correlate the placental *mir155* with the clinical parameters, periodontal pathogens including *Porphyromonas gingivalis, Tannerella forsythia, T. denticola, Fusobacterium nucleatum, and Prevotella intermedia* and viruses such as HSV, EBV, and HCMV subgingivally.

Our results were consistent with the study results of Dai Y et al. [38] who evidencedevidenced that LPS induces an increase in mir155 levels in pre-eclamptic pregnant women and stated that mir155 acts in part through the AP-1 and NF-κB inflammatory pathways. Additionally, *mir*-155 levels were expressed at a higher pace in the serum and placental samples of women with preeclampsia evidencing the presence of inflammation within placental tissues [39].

## 5. Conclusions

Overall, periodontal tissue destruction was seen in preeclamptic women with chronic periodontitis. From the present study findings, we advocated increased periodontal destruction, the presence of periodontal bacteria and viruses with the increased level of placental *mir*155 in preeclamptic women with chronic periodontitis. In our study, *mir*155 correlation with the periodontal parameters and periodontal pathogens further strengthen the evidence of periodontal inflammation as a risk of preeclampsia in pregnant women especially when associated with chronic periodontitis. *mir*155 can be considered to be one of the genetic biomarkers and can be used as a diagnostic tool for the early detection of PE. In the future, longitudinal interventional studies are needed to explore the function of treatment of periodontitis in decreasing the bacterial and viral load therefore reducing the further risk of PE.

## Figures and Tables

**Table 1:** Primer sequences of Putative Periodontopathic microorganisms bacteria (*Pg, Tf, Td, Pi,* and *Fn*), viruses (HSV, EBV, and HCMV), and MIR155.

Periodontapothic Microorganisms	Primer Sequence (5′–3′) FP (Forward Primer) RP (Reverse Primer)		Primer Length	Amplified Fragment Length (bp)
*Fusobacterium* *nucleatum*	ATTGTGGCTAAAAATTATAGTT ACCCTCACTTTGAGGATTATAG	(FP) (RP)	22 22	817
*Porphyromonas* *gingivalis*	AGGCAGCTTGCCATACTGCG ACTGTTAGCAACTACCGATGT	(FP) (RP)	20 21	404
*Prevotella intermedia*	CGTGGACCAAAGATTCATCGGTGGA CCGCTTTACTCCCCAACAAA	(FP) (RP)	26 20	259
*Tannerella forsythia*	GCGTATGTAACCTGCCCGCA TGCTTCAGTGTCAGTTATACCT	(FP) (RP)	21 22	641
*Treponema denticola*	TAATACCGAATGTGCTCATTTACAT TCAAAGAAGCATTCCCTCTTCTTCTTA	(FP) (RP)	26 27	316
Epstein-Barr virus inner	AGGCTGCCCACCCTGAGGAT GCCACCTGGCAGCCCTAAAG	(FP) (RP)	21 20	168
Herpes simplex virus inner	CAGTTCGGCGGTGCGGACAAA GCGTTTATCAACCGCACCTCC	(FP) (RP)	22 21	222
Human cytomegalovirus inner primer	AGTGTGGATGACCTACGGGCCATCG GGTGACACCAGAGAATCAGAGGAGC	(FP) (RP)	25 25	110
**MiRNA-155**	5‘-CTAGCCTGCAGGTATTCAAATATTTCCACAGA-3′ 5′-ATCCGGCCGGCCTGAAGATGGTTATGAACATA-3′,			(FP) (RP)
**U6-snRT**	5′-AAAATATGGAACGCTTCACGAATT-3′,			
**U6**	5′- CTCGCTTCGGCAGCACATATACT-3′ 5′- ACGCTTCACGAATTTGCGTGT-3′			(FP) (RP)

**Table 2:** PCR conditions and reaction set up for Bacteria and Viruses.

Periodontapothic Microorganism	Initial Denaturation	De Naturation	Annealing	Extension	Cycles	Final Extension
Fusobacterium nucleatum	94 °C FOR 5 min	94 °C FOR 30 s	55 °C FOR 30 s	72 °C FOR 30 s	30	72 °C FOR 3 min
Porphyromonas gingivalis	94 °C FOR 5 min	94 °C FOR 30 s	55 °C FOR 30 s	72 °C FOR 30 s	30	72 °C FOR 3 min
Prevotella intermedia	94 °C FOR 3 min	94 °C FOR 30 s	55 °C FOR 30 s	72 °C FOR 20 s	30	72 °C FOR 3 min
Tannerella forsythia	94 °C FOR 3 min	94 °C FOR 30 s	55 °C FOR 30 s	72 °C FOR 30 s	30	72 °C FOR 3 min
Treponema denticola	94 °C FOR 5 min	94 °C FOR 30 s	55 °C FOR 30 s	72 °C FOR 20 s	30	72 °C FOR 3 min
EBV INNER	94 °C FOR 3 min	94 °C FOR 30 s	63 °C FOR 15 s	72 °C FOR 30 s	30	72 °C FOR 3 min
Hsv INNER	94 °C FOR 1 min	94°C FOR 1 min	55 °C FOR 1 min	72 °C FOR 30 s	35	72 °C FOR 30 s
HCMV	94 °C FOR 5 min	94 °C FOR 30 s	59 °C FOR 30 s	72 °C FOR 30 s	25	72 °C FOR 3 min
MIR155	94 °C FOR 2 min	94 °C FOR 30 s	59 °C FOR 30 s	72 °C FOR 30 s	35	72 °C FOR 30 s

**Table 3:** Reaction setup for bacteria and viruses with final concentration.

Component	25 µL Reaction	Final Concentration
Luna Universal qPCR Master Mix	10 µL	1 X
Forward primer (10 µM)	1 µL	0.4 µM
Reverse primer (10 µM)	1 µL	0.4 µM
Template DNA	Variable	<100 ng
Nuclease-free Water	to 25 µL	

**Table 4:** Intergroup Comparison of demographic and periodontal variables in between the groups.

Variables	Controls	Group A	Group B	Group C	*p*-Value
Age	21.71 ± 1.92	22.14 ± 2.27	21.72 ± 1.95	22.21 ± 2.22	0.147 ^†^
Weight	57.01 ± 4.74	56.89 ± 4.52	56.83 ± 4.87	57.11 ± 4.85	0.975 ^†^
BMI	23.11 ± 2.89	24.99 ± 17.99	23.04 ± 2.51	24.46 ± 17.92	0.556 ^†^
SES	18.64 ± 5.21	19.39 ± 4.35	19.46 ± 4.27	19.69 ± 4.34	0.296 ^†^
DBP	78.61 ± 3.59	78.63 ± 3.69	93.00 ± 7.32	92.90 ± 7.15	<0.0001 *
SBP	118.15 ± 7.04	118.35 ± 6.97	144.78 ± 5.53	144.49 ± 5.70	<0.0001 *
Urine protein	16.87 ± 3.94	17.31 ± 3.85	35.11 ± 2.96	34.75 ± 320	<0.0001 *
PI	0.77 ± 0.22	1.62 ± 0.67	1.59 ± 0.67	1.86 ± 1.08	<0.0001 *
BOP	1.36 ± 0.59	4.07 ± 3.24	4.68 ± 5.49	1.00 ± 0.44	<0.0001 *
CAL	0.87 ± 0.23	8.25 ± 1.02	3.53 ± 1.05	0.87 ± 0.23	<0.0001 *
PPD	2.76 ± 0.63	5.21 ± 0.99	5.20 ± 0.98	2.79 ± 0.62	<0.0001 *

* —Significant, ^†^ —No Significant, *p*-value ≤ 0.05 considered statistically significant. Age, BMI expressed as Mean ± SD. BMI—Body mass index, socioeconomic status (SES), Diastolic blood pressure (DBP), Systolic blood pressure (SBP). Controls–systemically and periodontally healthy pregnant women. Group A–systemically healthy pregnant women with chronic periodontitis Group B–preeclamptic pregnant women with chronic periodontitis. Group C–preeclamptic pregnant women without chronic periodontitis.

**Table 5:** Comparison of the presence of bacteria and viruses in subgingival plaque and placental tissue samples in between the groups.

Periodontal Microorganisms		Controls		Group A		Group B		Group C		*p*-Value
		Mean ± SD	Range	Mean ± SD	Range	Mean ± SD	Range	Mean ± SD	Range	
*Tf*	Sub gingival Plaque	2.4 × 10^5^	(5.8 × 10^2^–4.24 × 10^6^)	4.26 × 10^6^	(2.4 × 10^3^–1.85 × 10^7^)	5.42 × 10^7^	(2.44 × 10^3^–6.41 × 10^8^)	5.25 × 10^6^	(2.2 × 10^2^–9.5 × 10^6^)	0.07 ^†^
	Placental tissue	2.6 × 10^5^	(5.4 × 10^2^–4.21 × 10^6^)	2.81 × 10^6^	(2.2 × 10^3^–1.5 × 10^7^)	4.25 × 10^7^	(2.44 × 10^3^–5.2 × 10^8^)	4.2 × 10^6^	(2.7 × 10^2^–5.2 × 10^6^)	0.07 ^†^
*Td*	Sub gingival Plaque	2.4 × 10^5^	(1.6 × 10^2^–5.2 × 10^6^)	4.2 × 10^6^	(2.8 × 10^2^–9.2 × 10^7^)	4.25 × 10^8^	(5.6 × 10^3^–8.62 × 1× 10^9^)	3.2 × 10^4^	(5.6 × 10^2^–4.25 × 10^6^)	0.062 ^†^
	Placental tissue	2.2 × 10^5^	(2.2 × 10^2^–5.5 × 10^6^)	4.6 × 10^6^	3.5 × 10^2^–7.8 × 10^7^)	5.25 × 10^8^	(7.9 × 10^3^–8.58 × 10^9^)	3.5 × 10^4^	(4.2 × 10^2^–8.8 × 10^6^)	0.045 *
*Pg*	Sub gingival Plaque	4.25 × 10^6^	(3.12 × 10^2^–6.52 × 10^7^)	5.92 × 10^6^	(1.59 × 10^2^–7.8 × 10^7^)	8.48 × 10^6^	(2.56 × 10^3^–5.8 × 10^9^)	4.22 × 10^5^	(5.8 × 10^2^–2.6 × 10^7^)	0.042 *
	Placental tissue	3.9 × 10^6^	(2.12 × 10^2^ –5.55 × 1× 10^7^)	5.54 × 10^6^	(2.45 × 10^2^–2.8 × 10^7^)	8.2 × 10^8^	(1.56 × 10^3^–4.1 × 10^9^)	5.15 × 10^5^	(4.2 × 10^2^–4.6 × 10^6^)	0.04 *
*Pi*	Sub gingival plaque	1.72 × 10^7^	(9.28 × 10^2^–3.23 × 10^8^)	7.24 × 10^7^	(1.82 × 10^4^–7.97 × 10^7^	7.52 × 10^8^	(1.86 × 10^5^–8.11 × 10^8^)	1.24 × 10^6^	(2.86 × 10^5^–4.2 × 10^6^)	0.005 *
	Placental tissue	1.6 × 10^5^	(2.6 × 10^2^–5.4 × 10^6^)	3.9 × 10^6^	(2.9 × 10^2^–2.4 × 10^7^)	5.58 × 10^6^	(54.5 × 10^3^–7.5 × 10^9^)	2.9 × 10^4^	(3.5 × 10^2^–4.25 × 10^6^)	0.05 *
*Fn*	Sub gingival plaque	1.29 × 10^4^	(4.52 × 10^2^–8.62 × 10^5^)	2.2 × 10^6^	(2.32 × 10^3^–4.22 × 10^7^)	7.22 × 10^8^	(2.44 × 10^3^–5.82 × 10^9^)	1.55 × 10^6^	(4.2 × 10^2^–8.48 × 10^6^)	0.045 *
	Placental tissue	2.22 × 10^4^	(3.45 × 10^2^–6.5 × 10^5^)	4.2 × 10^6^	(3.32 × 10^3^–2.27 × 10^7^)	6.52 × 10^8^	(2.54 × 10^3^–5.44 × 10^9^)	1.27 × 10^6^	(4.5 × 10^2^–8.6 × 10^6^)	0.065 ^†^
HSV	Sub gingival Plaque	4.8 × 10^6^	(5.5 × 10^4^–9.24 × 10^6^)	4.55 × 10^7^	(2.6 × 10^3^–4.8 × 10^8^)	4.9 × 10^7^	(4.4 × 10^3^–5.4 × 10^8^)	2.5 × 10^6^	(4.2 × 10^4^–9.5 × 10^7^)	0.072 ^†^
	Placental tissue	3.2 × 10^6^	(2.9 × 10^3^–5.5 × 10^7^)	7.6 × 10^8^	4.5 × 10^3^–4.8 × 10^9^)	8.2 × 10^9^	(8.7 × 10^4^–8.6 x10^10^)	2.5 × 10^6^	(2.2 × 10^2^–9.8 × 10^6^)	0.02 *
EBV	Sub gingival plaque	2.4 × 10^8^	(3.12 × 10^2^–6.52 × 10^7)^	5.22 × 10^7^	(2.87 × 10^3^–7.8 × 10^8^	8.7 × 10^7^	(2.42 × 10^4^–4.8 × 10^9^)	6.85 × 10^6^	(4.4 × 10^3^–5.2 × 10^7^)	0.004 *
	Placental tissue	4.2 × 10^6^	(5.6 × 10^3^–4.9 × 10^8^)	8.6 × 10^8^	(2.5 × 10^2^–7.2 × 10^9^)	7.4 × 1× 10^8^	(2.6 × 10^4^–5.5 × 10^9^)	2.6 × 10^6^	(4.5 × 10^4^–7.2 × 10^7^)	0.005 *
HCMV	Sub gingival plaque	4.25 × 10^8^	(6.22 × 10^3^–8.62 × 10^8^)	4.8 × 10^8^	(4.15 × 10^3^–4.11 × 10^9^)	6.85 × 10^6^	(4.58 × 10^4^–7.85 × 10^9^)	8.9 × 10^7^	(2.5 × 10^3^–4.48 × 10^7^)	0.042 *
	Placental tissue	3.25 × 10^6^	(4.2 × 10^2^–5.9 × 10^7^)	4.6 × 10^7^	(2.7 × 10^3^–1.5 × 10^8^)	7.8 × 10^8^	(2.6 × 10^3^–5.6 × 10^9^)	4.9 × 10^6^	(1.7 × 10^3^–5.5 × 10^7^)	0.03 *

* —Significant, ^†^ —No Significant. p-value ≤ 0.05 considered statistically significant. Tannerella forsythia (Tf), Treponema denticola (Td), Porphyromonas gingivalis (Pg), Prevotella intermedia (Pi), Fusobacterium nucleatum (Fn). Herpes Simplex Virus (HSV), Ebstein Barr Virus (EBV), and Human Cytomegalovirus (HCMV). Controls–systemically and periodontally healthy pregnant women. Group A–systemically healthy pregnant women with chronic periodontitis. Group B–preeclamptic pregnant women with chronic periodontitis. Group C–preeclamptic pregnant women without chronic periodontitis.

**Table 6:** Correlation of mir155 with Periodontal Microorganisms among Groups.

Microorganisms		Group 1	Group 2	Group 3	Group 4
		*mir155*	*mir155*	*mir155*	*mir155*
BOP	Corelation	0.013	–0.123	–0.079	–0.019
	*p*-value	0.004 *	0.255	0.626	0.821
PI	Corelation	–0.010	–0.199	–0.019	0.048
	*p*-value	0.513	0.160	0.313	0.030 *
PPD	Corelation	0.042	–0.042	0.066	–0.016
	*p*-value	0.037 *	0.510	0.033 *	0.638
CAL	Corelation	0.029	–0.014	0.008	–0.005
	*p*-value	0.410	0.489	0.045 *	0.950
*p g*	Corelation	–0.062	–0.054	–0.082	–0.054
	*p*-value	0.463	0.524	0.332	0.524
*T f*	Corelation	–0.008	–0.054	0.068	–0.080
	*p*-value	0.920	0.524	0.425	0.341
*T d*	Corelation	–0.052	–0.043	–0.043	–0.065
	*p*-value	0.538	0.613	0.613	0.440
*F n*	Corelation	–0.082	–0.042	0.051	–0.166
	*p*-value	0.332	0.619	0.045 *	0.047 *
*p i*	Corelation	–0.025	–0.082	–0.121	–0.082
	*p*-value	0.767	0.330	0.152	0.330
HSV	Corelation	0.072	0.073	0.076	0.071
	*p*-value	0.039 *	0.039 *	0.041 *	0.039 *
EBV	Corelation	0.052	0.133	0.054	–0.090
	*p*-value	0.536	0.112	0.322	0.285
HCMV	Corelation	0.027	0.027	0.027	0.072
	*p*-value	0.752	0.752	0.752	0.394

* —Significant. *p*-value ≤ 0.05 considered statistically significant. Tannerella forsythia (Tf), Treponema denticola (Td), Porphyromonas gingivalis (Pg), Prevotella intermedia (Pi), Fusobacterium nucleatum (Fn). Herpes Simplex Virus (HSV), Ebstein Barr Virus (EBV), and Human Cytomegalovirus (HCMV). Controls–systemically and periodontally healthy pregnant women. Group A–systemically healthy pregnant women with chronic periodontitis. Group B–preeclamptic pregnant women with chronic periodontitis. Group C–preeclamptic pregnant women without chronic periodontitis.

## References

[1] Agustín Zerón J. (1990). Glossary of periodontal terms. Rev. ADM.

[2] Chen C., Hemme C., Beleno J., Shi Z.J., Ning D., Qin Y., Tu Q., Jorgensen M., He Z., Wu L. (2018). Oral microbiota of periodontal health and disease and their changes after nonsurgical periodontal therapy. ISME J..

[3] Kesic L., Milasin J., Igic M.O.R. (2008). Microbial etiology of periodontal disease-Mini Review. Med. Biol..

[4] Offenbacher S., Jared H.L., O’Reilly P.G., Wells S.R., Salvi G.E., Lawrence H.P., Socransky S.S., Beck J.D. (1998). Potential pathogenic mechanisms of periodontitis associated pregnancy complications. Ann. Periodontol..

[5] Barak S., Oettinger-Barak O., Machtei E.E., Sprecher H., Ohel G. (2007). Evidence of periopathogenic microorganisms in placentas of women with preeclampsia. J. Periodontol..

[6] Han Y.W., Wang X. (2013). Mobile microbiome: Oral bacteria in extra-oral infections and inflammation. J. Dent. Res..

[7] Sadatmansouri S., Sedighpoor N., Aghaloo M. (2006). Effects of periodontal treatment phase I on birth term and birth weight. J. Indian Soc. Pedod. Prev. Dent..

[8] Reddy B.V.R., Tanneeru S., Chava V.K. (2014). The effect of phase-I periodontal therapy on pregnancy outcome in chronic periodontitis patients. J. Obstet. Gynaecol..

[9] Faraoni I., Antonetti F.R., Cardone J., Bonmassar E. (2009). miR-155 gene: A typical multifunctional microRNA. Biochim. Biophys. Acta.

[10] O’Connell R.M., Rao D.S., Baltimore D. (2012). microRNA regulation of inflammatory responses. Annu. Rev. Immunol..

[11] Peter S. (2004). Essentials of Prevention and Community Dentistry.

[12] Carranza F.A., Newman M.G., Takei H.H., Klokkevold P.R. (2006). Carranza’s Clinical Periodontology.

[13] Chomczynski P., Sacchi N. (1987). Single-step method of RNA isolation by acid guanidinium thiocyanate-phenol-chloroform extraction. Anal. Biochem..

[14] Jaiman G., Nayak P.A., Sharma S., Nagpal K. (2018). Maternal periodontal disease and preeclampsia in Jaipur population. J. Indian Soc. Periodontol..

[15] Wei B.-J., Chen Y.-J., Yu L., Wu B. (2013). Periodontal disease and risk of preeclampsia: A meta-analysis of observational studies. PLoS ONE.

[16] Mahendra J., Parthiban P.S., Mahendra L., Balakrishnan A., Shanmugam S., Junaid M., Romanos G.E. (2016). Evidence Linking the Role of Placental Expressions of Peroxisome Proliferator-Activated Receptor-γ and Nuclear Factor-Kappa B in the Pathogenesis of Preeclampsia Associated With Periodontitis. J. Periodontol..

[17] Jang H.C., Cho N.H., Min Y.K., Han I.K., Jung K.B., Metzger B.E. (1997). Increased macrosomia and perinatal morbidity independent of maternal obesity and advanced age in Korean women with GDM. Diabetes Care.

[18] Motedayen M., Rafiei M., Rezaei Tavirani M., Sayehmiri K., Dousti M. (2019). The relationship between body mass index and preeclampsia: A systematic review and meta-analysis. Int. J. Reprod. Biomed..

[19] Silva L.M., Coolman M., Steegers E.A., Jaddoe V.W., Moll H.A., Hofman A., Mackenbach J.P., Raat H. (2008). Low socioeconomic status is a risk factor for preeclampsia: The Generation R Study. J. Hypertens..

[20] Parikh N.I., Gonzalez J. (2017). Preeclampsia and Hypertension: Courting a Long While: Time to Make It Official. JAMA Intern. Med..

[21] Beck J.D., Papapanou P.N., Philips K.H., Offenbacher S. (2019). Periodontal Medicine: 100 Years of Progress. J. Dent. Res..

[22] Sugimoto H., Hamano Y., Charytan D., Cosgrove D., Kieran M., Sudhakar A., Kalluri R. (2003). Neutralization of circulating vascular endothelial growth factor (VEGF) by anti-VEGF antibodies and soluble VEGF receptor 1 (sFlt-1) induces proteinuria. J. Biol. Chem..

[23] Romero R., Avila C., Brekus C.A., Morotti R. (1991). The Role of Systemic and Intrauterine Infection in Preterm Parturition. Ann. N. Y. Acad. Sci..

[24] Han Y.W., Redline R.W., Li M., Yin L., Hill G.B., McCormick T.S. (2004). Fusobacterium nucleatum induces premature and term stillbirths in pregnant mice: Implication of oral bacteria in preterm birth. Infect. Immun..

[25] Amarasekara R., Jayasekara R.W., Senanayake H., Dissanayake V.H.W. (2015). Microbiome of the placenta in pre-eclampsia supports the role of bacteria in the multifactorial cause of pre-eclampsia. J. Obstet. Gynaecol. Res..

[26] Han Y.W., Fardini Y., Chen C., Iacampo K.G., Peraino V.A., Shamonki J.M., Redline R.W. (2010). Term stillbirth caused by oral Fusobacterium nucleatum. Obstet. Gynecol..

[27] Fardini Y., Wang X., Témoin S., Nithianantham S., Lee D., Shoham M., Han Y.W. (2011). Fusobacterium nucleatum adhesin FadA binds vascular endothelial cadherin and alters endothelial integrity. Mol. Microbiol..

[28] Cobb C.M., Kelly P.J., Williams K.B., Babbar S., Angolkar M., Derman R.J. (2017). The oral microbiome and adverse pregnancy outcomes. Int. J. Womens Health.

[29] Boggess K.A., Madianos P.N., Preisser J.S., Moise K.J., Offenbacher S. (2005). Chronic maternal and fetal Porphyromonas gingivalis exposure during pregnancy in rabbits. Am. J. Obstet. Gynecol..

[30] Ao M., Miyauchi M., Furusho H., Inubushi T., Kitagawa M., Nagasaki A., Sakamoto S., Kozai K., Takata T. (2015). Dental Infection of Porphyromonas gingivalis Induces Preterm Birth in Mice. PLoS ONE.

[31] Contreras A., Nowzari H., Slots J. (2000). Herpesviruses in periodontal pocket and gingival tissue specimens. Oral Microbiol. Immunol..

[32] Sharma S., Tapashetti R.P., Patil S.R., Kalra S.M., Bhat G.K., Guvva S. (2015). Revelation of Viral-Bacterial Interrelationship in Aggressive Periodontitis via Polymerase Chain Reaction: A Microbiological Study. J. Int. Oral Heal. JIOH.

[33] Sharma R., Padmalatha O., Kaarthikeyan G., Jayakumar N.D., Varghese S., Sherif K. (2012). Comparative analysis of presence of Cytomegalovirus (CMV) and Epsteinbarr virus -1 (EBV-1) in cases of chronic periodontitis and aggressive periodontitis with controls. Indian J. Dent. Res..

[34] Rustveld L.O., Kelsey S.F., Sharma R. (2008). Association between maternal infections and preeclampsia: A systematic review of epidemiologic studies. Matern. Child Health J..

[35] Silva N., Abusleme L., Bravo D., Dutzan N., Garcia-Sesnich J., Vernal R., Hernández M., Gamonal J. (2015). Host response mechanisms in periodontal diseases. J. Appl. Oral Sci..

[36] White D.W., Suzanne Beard R., Barton E.S. (2012). Immune modulation during latent herpesvirus infection. Immunol. Rev..

[37] Gao Z., Lv J., Wang M. (2017). Epstein-Barr virus is associated with periodontal diseases: A meta-analysis based on 21 case-control studies. Medicine.

[38] Dai Y., Diao Z., Sun H., Li R., Qiu Z., Hu Y. (2011). MicroRNA-155 is involved in the remodelling of human-trophoblast-derived HTR-8/SVneo cells induced by lipopolysaccharides. Hum. Reprod..

[39] Yang X., Zhang J., Ding Y. (2017). Association of microRNA-155, interleukin 17 A, and proteinuria in preeclampsia. Medicine.

[40] O’Connell R.M., Taganov K.D., Boldin M.P., Cheng G., Baltimore D. (2007). MicroRNA-155 is induced during the macrophage inflammatory response. Proc. Natl. Acad. Sci. USA.

[41] Lu H., Zhu C., Li F., Xu W., Tao D., Feng X. (2016). Putative periodontopathic bacteria and herpesviruses in pregnant women: A case-control study. Sci. Rep..

